# d‐Galactose induced early aging in human erythrocytes: Role of band 3 protein

**DOI:** 10.1002/jcp.30632

**Published:** 2021-11-15

**Authors:** Alessia Remigante, Sara Spinelli, Vincenzo Trichilo, Saverio Loddo, Antonio Sarikas, Michael Pusch, Silvia Dossena, Angela Marino, Rossana Morabito

**Affiliations:** ^1^ Biophysics Institute National Research Council Genova Italy; ^2^ Department of Chemical, Biological, Pharmaceutical, and Environmental Sciences University of Messina Messina Italy; ^3^ Department of Clinical and Experimental Medicine AOU Policlinico Universitario Messina Italy; ^4^ Institute of Pharmacology and Toxicology Paracelsus Medical University Salzburg Austria

**Keywords:** aging, anion exchange, band 3 protein, d‐galactose, erythrocytes, glycation, oxidative stress

## Abstract

Aging, a time‐dependent multifaceted process, affects both cell structure and function and involves oxidative stress as well as glycation. The present investigation focuses on the role of the band 3 protein (B3p), an anion exchanger essential to red cells homeostasis, in a d‐galactose ( d‐Gal)‐induced aging model. Anion exchange capability, measured by the rate constant of SO₄²^−^ uptake through B3p, levels of lipid peroxidation, oxidation of membrane sulfhydryl groups, B3p expression, methemoglobin, glycated hemoglobin (Hb), and the reduced glutathione/oxidized glutathione ratio were determined after exposure of human erythrocytes to 25, 35, 50, and 100 mmol/L d‐Gal for 24 h. Our results show that: (i) in vitro application of d‐Gal is useful to model early aging in human erythrocytes; (ii) assessment of B3p ion transport function is a sensitive tool to monitor aging development; (iii) d‐Gal leads to Hb glycation and produces substantial changes on the endogenous antioxidant system; (iv) the impact of aging on B3p function proceeds through steps, first involving Hb glycation and then oxidative events at the membrane level. These findings offer a useful tool to understand the mechanisms of aging in human erythrocytes and propose B3p as a possible target for new therapeutic strategies to counteract age‐related disturbances.

## 1 INTRODUCTION

Aging is a complex and progressive physiological alteration of the organism characterized by the accumulation of degenerative processes, which ultimately compromise cell and tissue function (Wagner et al., 2016). As such, aging is the main risk factor for almost all chronic diseases, including cardiovascular and neurological diseases, cancer, and diabetes (Calcinotto et al., 2019; Ferrera et al., 2021; Guzik & Cosentino, 2018; Kritsilis et al., 2018; La Russa et al., 2020). The processes that contribute to aging and the development of age‐associated diseases comprise DNA damage, oxidative stress, alterations in the cell redox system, glycation events, and apoptosis (Franzke et al., 2015; Hegab et al., 2012; Luevano‐Contreras & Chapman‐Novakofski, 2010; Simon et al., 2000). In spite of many theories of aging, as yet none has been able to fully explain the mechanisms that drive its main processes. Among the experimental models of aging, long‐term d‐galactose (d‐Gal) exposure is the most similar to natural aging (Azman & Zakaria, 2019). This phenomenon is not related to the galactosemia condition. In fact, d‐Gal is a reducing sugar, whose abnormally increased levels can be converted into d‐galacto‐exodialdose and hydroperoxide by the galactose oxidase enzyme, thus resulting in the generation of reactive oxygen species (ROS) and oxidative stress. Alternatively, d‐Gal can initiate nonenzymatic glycation reactions with free amino acid groups to form advanced glycation endproducts (AGEs) (Luevano‐Contreras & Chapman‐Novakofski, 2010). Among the many cellular models used to investigate the biochemical alterations during aging, as well as the impact of oxidative stress, erythrocytes offer key advantages (Aminoff et al., 1992; Pandey & Rizvi, 2010). Red blood cells play vital roles in many physiological and metabolic processes. The abundance of polyunsaturated fatty acids in the cell membrane and the continuous exposure to circulating ROS make erythrocytes a primary target of oxidative stress (Abdallah et al., 2011). In physiological conditions, erythrocytes possess endogenous mechanisms, that is, many antioxidant enzymes, to effectively defend against intracellular oxidative stress (Morabito et al., 2017; Remigante et al., 2021). However, the rate of oxidative damage may increase during pathological conditions (de Franceschi et al., 2013; Pantaleo et al., 2016; Shan et al., 2016), and an increase of oxidative stress has been linked to a shortening of erythrocytes life span (Rizvi et al., 2011).

During natural aging, erythrocyte volume decreases with time, and this process is accompanied by an increase in density and a decrease in hemoglobin content. These changes are related to a loss of plasma membrane constituents, including cholesterol and phospholipids, and a progressive decrease in the mean surface area (Buehler & Alayash, 2005). It has also been shown that aging has a strong impact on band 3 protein (B3p) (Stevenson et al., 2017). B3p is an integral membrane protein and acts as an anchor able to connect the soluble cytoplasmic proteins and the components of the cytoskeleton to the cell membrane (Arakawa et al., 2015; Reithmeier et al., 2016). Consequently, degradation of B3p alters the link between the cytoskeleton and the lipid bilayer, thus leading to an impairment of erythrocyte functionality (Koshkaryev et al., 2020). In addition, oxidative damage induces an abnormal erythrocyte morphology and increased susceptibility to osmotic and mechanical stress, as well as alterations of ion homeostasis, which is closely related to the chloride/bicarbonate exchange activity of B3p (Bosman, 2018; Morabito et al., 2016). In this regard, the anion exchange capability of B3p can be estimated by measuring the uptake of SO_4_^2−^, which is slower and easier to detect compared to the uptake of bicarbonate or chloride (Jennings, 1976).

Recently, erythrocytes have been considered as a valid model to investigate the impact of oxidative damage on human health. The anion exchange capability of B3p has been recognized as a sensitive tool to study the effect of oxidative stress, oxidative stress‐related diseases, and high glucose levels on membrane transport physiology (Morabito et al., 2020; Remigante et al., 2019). On the basis of these considerations, the hypothesis of the present study is that aging could affect the functional role of B3p. Exposure to high d‐Gal concentrations (25–100 mmol/L) was used here for the first time to induce erythrocyte aging. As it is widely demonstrated that aging is associated with increased oxidative stress as well as glycation events (Moldogazieva et al., 2019), both processes were investigated. Considering that red cells are continually exposed to oxidizing molecules transported within the vascular system, we suggest that the B3p assessment could be a good tool to detect the early progression of diseases linked to aging.

## 2 MATERIALS AND METHODS

### 2.1 Solutions and chemicals

Chemicals were purchased from Sigma‐Aldrich. d‐Gal was freshly prepared in H_2_O and diluted from a 1 mmol/L stock solution. 4,4ʹ‐diisothiocyanatostilbene‐2,2ʹ‐disulfonate (DIDS) stock solution (10 mmol/L) was prepared in dimethyl sulfoxide (DMSO). Thiobarbituric acid (TBA) was freshly prepared in 0.05 M NaOH. *N*‐ethylmaleimide (NEM) was prepared in ethanol and diluted starting from a 310 mmol/L stock solution. 5,5‐dithio‐bis‐(2‐nitrobenzoic acid) (DTNB) stock solution (50 mmol/L) was prepared in ethanol. The specific catalase inhibitor 3‐amino‐1,2,4‐triazole (3‐AT) (Margoliash et al., 1960), freshly prepared H_2_O_2_ and NaNO_2_ were dissolved in distilled water and diluted from, respectively, 3 M, 50 mmol/L, and 30% w/w stock solutions. DMSO and ethanol were previously tested on RBC at their final concentration to exclude any damage related to the solvent.

### 2.2 Erythrocyte preparation

Human whole blood was obtained from healthy volunteers, upon informed consent, with a plasma concentration of glycated hemoglobin (A1c) less than 5%. Blood samples, collected in tubes containing ethylenediaminetetraacetic acid (EDTA) as an anticoagulant, were washed three times in isotonic solution (composition: 145 mmol/L NaCl, 5 mmol/L 4‐(2‐hydroxyethyl)−1‐piperazineethanesulfonic acid (HEPES), 5 mmol/L glucose; pH 7.4, osmotic pressure: 300 mOsm/kgH_2_O) and centrifuged (1200*g*, 5 min, Thermo Fisher Scientific) to remove both plasma and buffy coat. Subsequently, red cells were suspended at different hematocrit concentrations in an isotonic solution or, alternatively, in a d‐Gal‐containing solution and directed to several experimental tests. After each treatment, samples were subjected to SO_4_^2−^ uptake measurement, oxidative condition assessment, Western blot analysis, and glycated hemoglobin determination.

### 2.3 SO_4_^2−^ uptake measurement

#### 2.3.1 Control condition

SO_4_^2−^ uptake through B3p was detected as described elsewhere (Romano & Passow, 1984; Romano et al., 1998). After washing, red cell samples were suspended to 3% hematocrit and addressed to control conditions assessment. Successively, red cells were suspended in 35 ml of an SO_4_^2−^‐containing isotonic solution henceforth referred to as SO_4_^2−^ medium (composition: Na_2_SO_4_ 118 mmol/L, HEPES 10 mmol/L, glucose 5 mmol/L, pH 7.4, osmotic pressure 300 mOsm/kgH_2_O) and incubated at 25°C. At specific time intervals (5, 10, 15, 30, 45, 60, 90, and 120 min), 5 ml erythrocyte suspension was transferred in a tube containing DIDS (10 μM) to block B3p activity (Jessen et al., 1986), and kept on ice. After treatment with DIDS, samples were washed three times by centrifugation (4°C, 1200*g*, 5 min; Thermo Fisher Scientific), resuspended in isotonic solution to wash SO_4_^2−^ from the external medium, and then lysed by distilled water (1 ml). All proteins were precipitated by treatment with perchloric acid (4% v/v). After centrifugation (4°C, 2500*g*, 10 min; Thermo Fisher Scientific), the supernatant containing SO_4_^2−^ was addressed to turbidimetric analysis. SO_4_^2−^ precipitation was detected by mixing the following components: 500 μl supernatant from each sample, 500 μl glycerol (diluted in distilled water, 1:1), 1 ml 4 M NaCl plus 37% hydrochloric acid (HCl) solution (12:1) and, finally, 500 μl 1.24 M BaCl_2_•2H_2_O. Then, the absorbance of samples was measured at 425 nm using a spectrophotometer (BioPhotometer Plus; Eppendorf). A calibrated standard curve, obtained by precipitating known SO_4_^2−^ concentrations, was used to convert the absorbance to [SO_4_^2−^] L cells × 10^−2^. Moreover, the rate constant of SO_4_^2−^ uptake (min^−1^) was estimated by the following equation: *C_t_* = *C*_∞_ (1 − *e* ^−*rt*^) + *C*_0_, where *C_t_, C*_∞_, and *C*_0_ stand for the intracellular SO_4_^2−^ concentrations at time *t*, 0, and ∞, respectively, *e* is Neper number (2.7182818), *r* is the rate constant accounting for the process velocity, and *t* is the time of each sample withdrawal. The rate constant represents the inverse of the time needed to achieve ~63% of final SO_4_^2−^ intracellular concentration (Romano et al., 1998) and [SO_4_^2−^] L cells × 10^−2^ reported in figures stands for SO_4_^2−^ micromolar concentration trapped by 5 ml red cells (3% hematocrit).

#### 2.3.2 d‐Gal‐treated erythrocytes

Once washed and suspended to 3% hematocrit and after 24 h incubation at different d‐Gal concentrations added to the isotonic solution (25, 35, 50, and 100 mmol/L, at 25°C), red cell samples were further washed and centrifuged (4°C, 1200*g*, 5 min; Thermo Fisher Scientific) to substitute the supernatant with SO_4_^2−^ medium. The rate constant of SO_4_^2−^ uptake was successively determined according to what was described for the control condition.

### 2.4 Thiobarbituric‐acid‐reactive substances (TBARS) levels measurement

TBARS result from the reaction between thiobarbituric acid (TBA) and malondialdehyde, the end product of lipid peroxidation (Almroth et al., 2005). To test whether d‐Gal induced oxidative damage at the lipid level, the protocol proposed by Mendanha et al. (2012) was performed, with slight modifications. Briefly, after d‐Gal incubation at different concentrations (25, 35, 50, and 100 mmol/L), red cells were centrifuged (1200*g*, 5 min; Thermo Fisher Scientific) and suspended at 20% hematocrit. 1.5 ml of red cells were treated with 10% (w/v) trichloroacetic acid. Next, red cell samples were centrifuged (3000*g*, 10 min; Thermo Fisher Scientific) and 1 ml of TBA (1% in 0.05 M NaOH) was added to the supernatant. The mixture was heated and kept at 95°C for 30 min. Finally, TBARS levels were obtained by subtracting 20% of the absorbance at 453 nm from the absorbance at 532 nm (BioPhotometer Plus; Eppendorf). In parallel, some erythrocyte samples were incubated with 10 mmol/L H₂O₂, for 1 h at 25°C (positive control), as this compound is known to induce a strong lipid peroxidative effect (Sokolowska et al., 1999). Results are indicated as μM TBARS levels (1.56 × 10^5^ M^−1^ cm^−1^ molar extinction coefficient).

### 2.5 Membrane sulfhydryl (–SH) group content measurement

Measurement of –SH groups was performed according to Aksenov and Markesbery (2001), with some modifications. Shortly, after 24 h of incubation in d‐Gal‐containing solutions, red cell samples (100 μl) were centrifuged (1200*g*, 5 min; Thermo Fisher Scientific) and resuspended to 35% hematocrit. One milliliter of distilled water was added, and a 50‐μl aliquot of this mixture was diluted in 1 ml of phosphate‐buffered saline (pH 7.4) containing 1 mmol/L EDTA. Thirty microliters of 10 mmol/L DTNB were added to start the reaction and the samples were incubated for 30 min in a dark room at 25°C. Control samples, without proteins or DTNB, were simultaneously handled. After a 30 min incubation at 25°C, samples were spectrophotometrically measured at 412 nm (BioPhotometer Plus; Eppendorf), and TNB levels were determined by comparison to blank (DTNB absorbance). Incubation with 2 mmol/L NEM for 1 h at 25°C (positive control) was used to obtain complete oxidation of membrane –SH group (Morabito et al., 2015, 2016). Results are reported as μM TNB/mg protein and data were normalized to protein content.

### 2.6 Preparation of erythrocyte membranes

Erythrocyte membrane preparation was detected as described by other authors (Pantaleo et al., 2016), with slight modifications. Briefly, after incubation with d‐Gal, packed red cells were diluted into 1.5 ml of cold hemolysis solution (2.5 mM NaH_2_PO_4_) added with a cocktail of protease inhibitors (1 mmol/L phenylmethylsulfonyl fluoride, 1 mmol/L NaF, and 1 mmol/L Na_3_VO_4_). Samples were centrifuged several times (18,000*g*, 10 min, 4°C; Eppendorf) to remove hemoglobin. The membranes obtained were solubilized by 1% (v/v) sodium dodecyl sulfate (SDS) and kept on ice for 20 min. After centrifugation (13,000*g*, 30 min, at 4°C; Eppendorf), the supernatant containing the solubilized membrane proteins was stored at −80°C and used for determination of protein content (Bradford, 1976).

#### 2.6.1 SDS‐polyacrylamide gel electrophoresis (PAGE) preparation and Western blot analysis

Membrane proteins were solubilized in Laemmli buffer (Laemmli, 1970) in a volume ratio of 1:1 and kept at 95°C for 10 min. The protein samples (20 μl) were separated by 7.5% (w/v) SDS‐PAGE and transferred to polyvinylidene fluoride membrane by applying a constant voltage (75 V) at 4°C for 2 h. Membranes were blocked in 5% bovine serum albumin diluted in Tris‐buffered saline (composition: 150 mmol/L NaCl and 15 mmol/L Tris‐HCl) containing 0.1% (v/v) Tween‐20 (TBST) for 1 h at room temperature, and incubated overnight at 4°C with a monoclonal anti‐B3p antibody (B9277; Sigma‐Aldrich), produced in mouse and diluted 1:5000 in TBST. Successively, membranes were incubated for 1 h at room temperature with peroxidase‐conjugated goat anti‐mouse immunoglobulin G secondary antibodies (A9044; Sigma‐Aldrich), diluted 1:10,000 in TBST. To confirm the presence of equal amounts of proteins, a mouse monoclonal anti‐actin antibody (A1978; Sigma‐Aldrich) diluted 1:1000 in TBST, was incubated with the same membrane, according to Yeung and Stanley (2009). A chemiluminescence detection system (Super Signal West Pico Chemiluminescent Substrate; Pierce Thermo Scientific) was employed to detect signals, and the images were imported to analysis software (v2003; ImageQuant TL). The intensity of the corresponding protein bands was determined by densitometry (ChemiDoc™ XRS*+*; Bio‐Rad).

### 2.7 Determination of methemoglobin (MetHb) levels

MetHb levels were determined as reported by Naoum & Magaly da Silva (2004), with slight modifications. The proposed assay is based on MetHb and (oxy)‐hemoglobin (Hb) determination by spectrophotometry at two specific wavelengths, 630 and 540 nm, respectively. After incubation in d‐Gal, 25 μl of erythrocytes at 35% hematocrit were lysed in 1975 μl hypotonic buffer (composition: 2.5 mmol/L NaH_2_PO_4_, pH 7.4; 4°C). Then, samples were centrifuged (13,000*g*, 15 min, 4°C; Eppendorf) to eliminate membranes. The absorbance of the supernatant was measured (BioPhotometer Plus; Eppendorf). Incubation with 4 mmol/L NaNO_2_ (for 1 h at 25°C), a well‐known MetHb‐forming agent, was used to obtain complete Hb oxidation (Zavodnik et al., 1999). MetHb percentage (%) was determined as follows: % MetHb = (OD630/OD540) × 100 (OD is optical density).

### 2.8 Measurement of glycated Hb (%A1c)

The glycated Hb content (%A1c) was determined with the A1c liquidirect reagent as previously described by Sompong et al. (2015), with slight modifications. Briefly, after incubation with d‐Gal samples were lysed in hypotonic buffer and then incubated with latex reagent at 37°C for 5 min. The samples were spectrophotometrically measured at 610 nm (BioPhotometer Plus; Eppendorf). The A1c content, expressed as a percentage, was calculated from a standard curve constructed by using known A1c concentrations.

### 2.9 Measurement of reduced glutathione (GSH) content

GSH levels were assayed according to Giustarini et al. (2013), with slight modifications. This assay is based on the oxidation of GSH by Ellman's reagent DTNB, which produces oxidized glutathione (GSSG) and 3‐thio‐2‐nitro‐benzoic acid (TNB), absorbing at a wavelength of 412 nm. After incubation with d‐Gal, the content of GSH was measured by Cayman's GSH assay kit by an enzymatic recycling method with glutathione reductase (Teti et al., 2005). The amount of GSSG was calculated by the following formula: 1/2 GSSG = GSH_total_ − GSH_reduced_. Results are expressed as a GSH/GSSG ratio (Morabito et al., 2020).

### 2.10 Experimental data and statistics

Data are represented as arithmetic means ± SEM. For statistical analysis and graphics, GraphPad Prism (version 6.0; Windows) and Excel (Windows; Microsoft) software were used. Significant differences between mean values were determined by one‐way analysis of variance, followed by Dunnet's multiple comparison posttest. Statistically significant differences were determined at *p < 0.05, **p < 0.01, ***p < 0.001; *N* corresponds to the number of independent measurements.

## 3 RESULTS

### 3.1 SO_4_^2−^ uptake measurement

**Figure 1:**
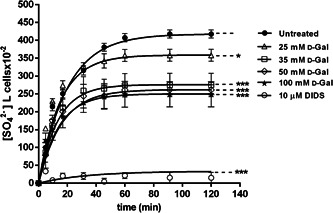
Time course of SO_4_^2−^ uptake in untreated (control, N = 12) and d‐Gal (d‐galactose)‐treated erythrocytes (*N* = 7) and in the presence of 10 µmol/L DIDS (4,4ʹ‐diisothiocyanatostilbene‐2,2ʹ‐disulfonate) (*n* = 5). *p < 0.05 and ***p < 0.001 versus control, as determined by one‐way analysis of variance followed by Dunnett's multiple comparisons posthoc test

Figure 1 illustrates SO_4_^2−^ uptake as a function of time in untreated (control) and 25, 35, 50, or 100 mmol/L d‐Gal‐treated erythrocytes, after 24 h of incubation. In control conditions, SO_4_^2−^ uptake progressively increased and reached equilibrium within 45 min (rate constant of SO_4_^2−^ uptake = 0.050 ± 0.001 min^−1^). The rate constant in 25 mmol/L d‐Gal (0.067 ± 0.001 min^−1^) was slightly, but significantly different with respect to control (*p < 0.05). Similarly, in 35, 50, or 100 mmol/L d‐Gal‐treated erythrocytes, the rate constant was significantly higher than control (Table 1). SO_4_^2−^ uptake was almost completely blocked by 10 µmol/L DIDS, applied at the beginning of incubation in SO_4_^2−^ medium (0.017 ± 0.001 min^−1^, ***p < 0.001, Table 1), consistent with an uptake via B3p. More importantly than the rate constant per se, SO_4_^2−^ amounts internalized at 45 min by d‐Gal‐treated erythrocytes were progressively smaller with increasing d‐Gal concentration (Table 1) (***p < 0.001, Table 1). In DIDS‐treated cells, intracellular SO_4_^2−^ amount after 45 min of incubation in SO_4_^2−^ medium (4.86 ± 5.50) was significantly lower than that determined in both control or in d‐Gal‐treated erythrocytes (***p < 0.001, Table 1).

**Table 1:** Rate constant of SO_4_^2−^ uptake and amount of SO_4_^2−^ trapped in untreated (control) and in d‐Gal‐treated erythrocytes

	Rate constant (min^−1^)	Time (min)	*N*	SO_4_^2−^ amount trapped after 45 min of incubation in SO_4_^2−^ medium [SO_4_^2−^] L cells × 10^−2^
Untreated (control)	0.050 ± 0.001	19.86	12	380.85 ± 15.19
25 mmol/L d‐Gal	0.067 ± 0.001*	14.79	7	342.35 ± 35.64*
35 mmol/L d‐Gal	0.075 ± 0.001***	13.22	7	264.85 ± 37.24***
50 mmol/L d‐Gal	0.063 ± 0.001***	15.67	7	244.35 ± 21.90***
100 mmol/L d‐Gal	0.071 ± 0.001***	13.60	7	241.35 ± 16.43***
10 µmol/L DIDS	0.017 ± 0.001***	58.82	5	4.86 ± 5.50***

*Note*: Data are presented as means ± SEM from separate experiments (*N*), where *p < 0.05 and ***p < 0.001 versus control, as determined by one‐way analysis of variance followed by Dunnett's multiple comparisons posthoc test.

Abbreviations: DIDS, 4,4ʹ‐diisothiocyanatostilbene‐2,2ʹ‐disulfonate; d‐Gal, d‐galactose.

### 3.2 TBARS levels

**Figure 2:**
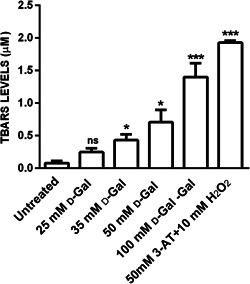
TBARS levels (µ mmol/L) in untreated erythrocytes (control) and in erythrocytes treated for 24 h with d‐Gal or alternatively with 50 mmol/L 3‐AT (preincubation for 10 min) + 10 mmol/L H_2_O_2_ for 1 h. d‐Gal, d‐Galactose; ns, not significant versus untreated; TBARS, thiobarbituric‐acid‐reactive substances. *p < 0.05 and ***p < 0.001 versus control, as determined by one‐way analysis of variance followed by Dunnett's multiple comparisons posthoc test (*N* = 7)

TBARS levels measured in d‐Gal‐treated erythrocytes after 24 h incubation are reported in Figure 2. After treatment with 25 mmol/L d‐Gal, TBARS levels were not significantly different with respect to those of untreated red cells. In erythrocytes treated with 35, 50, or 100 mmol/L d‐Gal and in erythrocytes treated with 50 mmol/L 3‐AT* + *10 mmol/L H_2_O_2_ (positive control), TBARS levels were significantly higher than those measured in untreated erythrocytes. These findings suggest that strong oxidation of membrane lipids occurred.

### 3.3 Membrane –SH group content measurement

**Figure 3:**
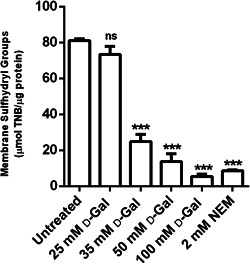
Membrane sulfhydryl group content (µmol TNB/µg protein) in untreated erythrocytes (control) and in erythrocytes treated with d‐Gal for 24 h or NEM for 1 h. d‐Gal, d‐Galactose; NEM, *N*‐ethylmaleimide; ns, not significant versus control; TNB, 3‐thio‐2‐nitro‐benzoic acid. ***p < 0.001 versus control, as determined by one‐way analysis of variance followed by Dunnett's multiple comparisons posthoc test (*N* = 6)

Figure 3 shows the membrane –SH group content (µmol/L TNB/µg protein) of red cells treated with the oxidizing compound NEM (2 mmol/L) for 1 h or increasing concentrations of d‐Gal for 24 h. As expected, exposure to NEM led to a significant reduction in membrane –SH group content. Membrane –SH group in 25 mmol/L d‐Gal‐treated erythrocytes were not significantly different with respect to control (untreated erythrocytes). Conversely, in 35, 50, and 100 mmol/L d‐Gal‐treated erythrocytes, membrane –SH group abundance was significantly reduced when compared to that measured in control conditions, thus denoting that strong oxidation of cellular proteins occurred.

### 3.4 B3p expression levels

**Figure 4:**
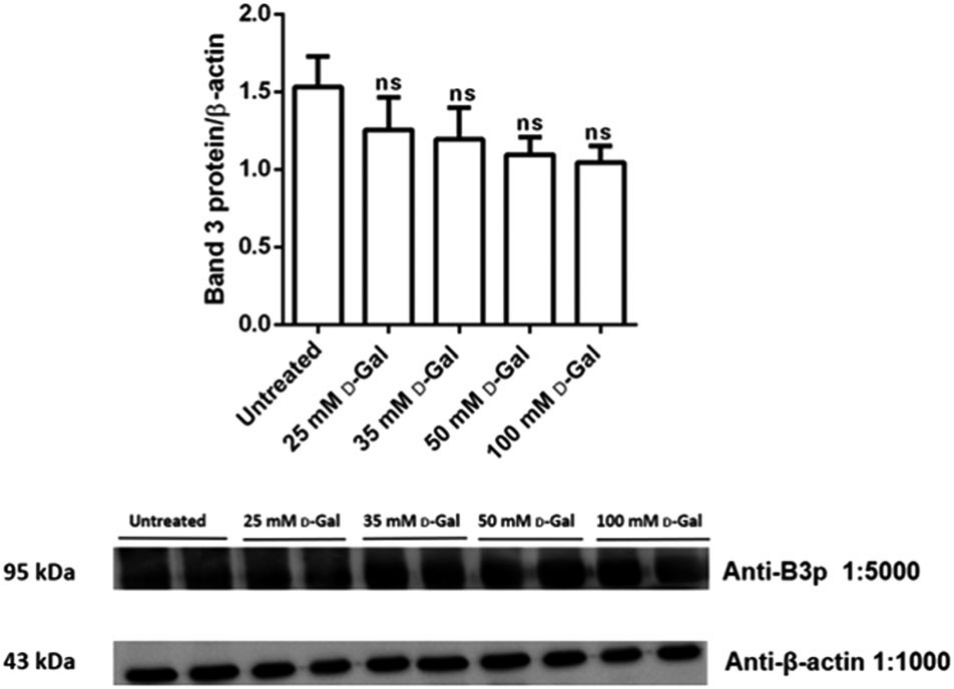
Band 3 protein expression levels measured in untreated (control) and in 25, 35, 50, and 100 mol/L d‐Gal (d‐galactose)‐treated (24 h) erythrocytes, detected by Western blot analysis. ns, not significant versus untreated (control), as determined by one‐way analysis of variance followed by Dunnett's multiple comparisons posthoc test (*N* = 5)

Figure 4 shows B3p expression levels in erythrocytes incubated with increasing concentrations of d‐Gal for 24 h. B3p expression levels were not significantly different with respect to those determined in control erythrocytes.

### 3.5 MetHb level

**Figure 5:**
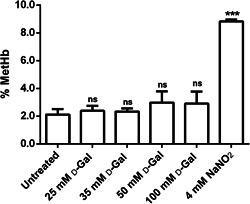
Methemoglobin (MetHb) levels (%) measured in untreated erythrocytes (control) and in erythrocytes treated with d‐Gal (d‐galactose) for 24 h or 4 mmol/L NaNO_2_ for 1 h. ns, not significant versus control and 4 mmol/L NaNO_2_. ***p < 0.001 versus control as determined by one‐way analysis of variance followed by Dunnett's multiple comparisons posthoc test (*N* = 8)

Figure 5 shows MetHb levels (% MetHb) in red cells treated with the well‐known MetHb‐forming agent NaNO_2_ (4 mmol/L) for 1 h, or different d‐Gal concentrations for 24 h. As expected, after exposure to NaNO_2_, MetHb levels (%) were significantly higher than those measured in untreated erythrocytes (control). In contrast, following treatment with d‐Gal, MetHb levels were not significantly different with respect to those detected in control.

### 3.6 Glycated Hb levels

**Figure 6:**
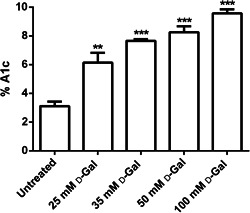
Glycated hemoglobin content (% A1c) in erythrocytes incubated for 24 h with different d‐Gal (d‐galactose) concentrations (25, 35, 50, and 100 mmol/L). **p < 0.01 and ***p < 0.001 versus untreated (control), as determined by one‐way analysis of variuance followed by Dunnett's multiple comparisons posthoc test (*N* = 10)

Figure 6 shows the glycated Hb levels (% A1c) measured in erythrocytes treated with increasing concentrations of d‐Gal for 24 h. The %A1c levels measured following d‐Gal exposure were significantly increased with respect to those of the control (untreated erythrocytes).

### 3.7 GSH/GSSG ratio measurement

**Figure 7:**
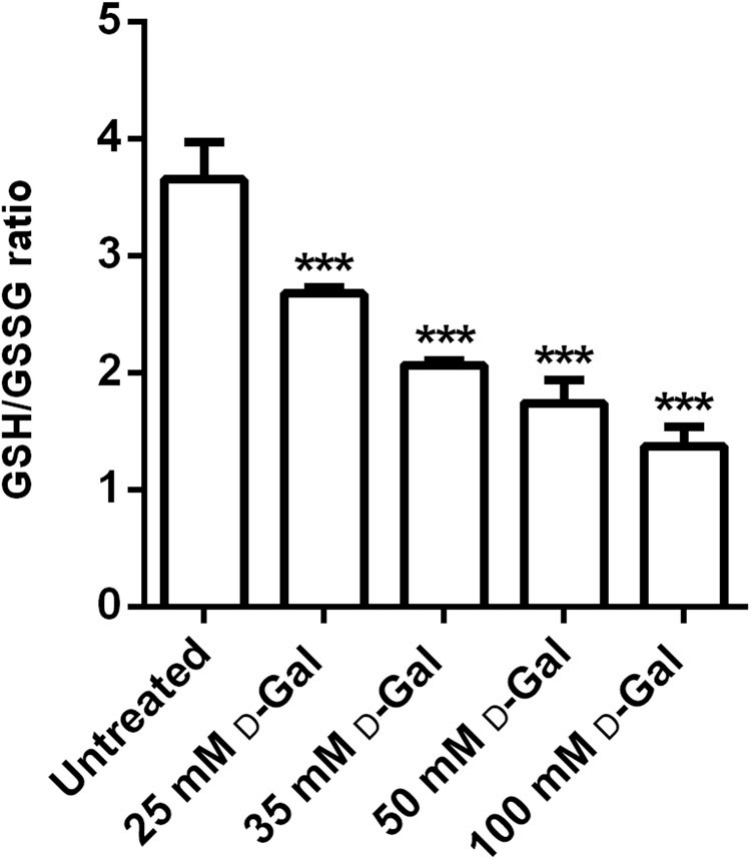
Estimation of the GSH/GSSG ratio measured in erythrocytes incubated for 24 h with different concentrations (25, 35, 50, and 100 mmol/L) of d‐Gal (d‐galactose). GSH, reduced glutathione; GSSG, oxidized glutathione. ***p < 0.001 versus untreated (control) erythrocytes, determined by one‐way analysis of variance followed by Dunnett's multiple comparisons posthoc test (*N* = 8)

Figure 7 shows the GSH/GSSG ratio measured in d‐Gal‐treated erythrocytes. The GSH/GSSG ratio measured after a 24 h incubation with d‐Gal was significantly lower with respect to that detected in control erythrocytes. This effect can be associated with an increased GSSG abundance and/or decreased GSH concentration, both indicative of cellular oxidative stress.

## 4 DISCUSSION

Aging is a natural phenomenon that occurs in all cells, tissues, and organs of the body (Wagner et al., 2016). Several studies suggest that chronic administration of d‐Gal is an appropriate model to study the effects of aging on human health (Azman & Zakaria, 2019; Park et al., 2020). It has been widely demonstrated that aging is mediated by oxidative and glycation events, potential conformational changes in protein structure, increased cell density, and reduced cell volume (Ansari & Dash, 2013; Bo‐Htay et al., 2018). All of these factors can also contribute to inducing biochemical, physical, and structural alterations in red blood cells, with specific regard to B3p (Stevenson et al., 2017). However, the effects of high concentrations of d‐Gal on human erythrocytes and the use of erythrocytes as a model of aging are still poorly investigated. Erythrocytes are peculiar cells lacking a nucleus but equipped with “high‐performance” proteins ensuring their homeostasis in oxygenated/deoxygenated medium and, in turn, body survival. Among erythrocyte proteins, the anion exchanger B3p involved in oxygen transport, ion balance, and cell shape maintenance has received the attention of researchers for years, mostly to monitor the impact of oxidative conditions on these cells (Matte et al., 2014; Pantaleo et al., 2016; Remigante et al., 2019). The present study focused on B3p function after d‐Gal treatment. In particular, the purpose was to investigate the impact of d‐Gal‐induced aging on one of the B3p functions, that is, the anion exchange capability, and in parallel to verify the potential mechanisms linked to aging through which high d‐Gal doses could affect this function.

The first aim was to measure the SO_4_^2−^ uptake through B3p (Morabito et al., 2016) after a 24‐h treatment with increasing concentrations (25, 35, 50, and 100 mmol/L) of d‐Gal. In these experimental conditions, the rate constant for SO_4_^2−^ uptake was accelerated and, in parallel, SO_4_^2−^ uptake was significantly reduced (Figure 1 and Table 1). This finding appears to be in disagreement with what was reported by our group in a recent study (Remigante et al., 2020), where the exposure of erythrocytes to 10 mmol/L d‐Gal for 24 h has been observed to induce a reduction (0.051 ± 0.001 in 19.60 min) of the rate constant for SO_4_^2−^ uptake, not accompanied by lipid peroxidation, oxidation of –SH membrane groups, alteration of GSH/GSSG ratio, or MetHb formation (Remigante et al., 2020). Therefore, it is not surprising that this different effect on the SO_4_^2−^ transport kinetics observed in the present study could be most likely linked to other mechanisms than oxidative stress, putatively glycated Hb formation.

The evidence that B3p exhibits modifications in the rate constant for SO_4_^2−^ uptake following exposure of human erythrocytes to oxidative stress has been already demonstrated. In particular, H_2_O_2_‐induced oxidative stress provoked a reduction in the rate constant for SO_4_^2−^ uptake (Morabito et al., 2016), whereas treatment with high glucose induced an acceleration of anion exchange (Morabito et al., 2020). Therefore, it is tempting to speculate that such a two‐sided effect on anion exchange velocity depends on the specific structure targeted by the stressors and on the possible underlying pathways.

Similar to what was performed in the previous investigation (Remigante et al., 2020) and to better explain the altered functional mechanism observed in the present experiments, lipid peroxidation and oxidation of membrane –SH groups, mainly belonging to B3p (Roy et al., 2005) have been evaluated. In these conditions, the use of 35, 50, and 100 mmol/L d‐Gal produced significant oxidation of both lipids, and sulphydryl groups (Figures 2 and 3) of membrane proteins, mainly belonging to B3p, present in one million copies upon erythrocytes membrane (Reithmeier et al., 2016). On the contrary, 25 mmol/L **d**‐Gal dose was not sufficient to induce substantial oxidative change on erythrocytes membrane, though able to cause a functional alteration of B3p, demonstrating an increased rate of anion exchange but reduced SO_4_^2−^ uptake. Importantly, higher d‐Gal concentrations (35, 50, and 100 mmol/L) produced consistent effects, including increased TBARS and decreased total antioxidant capacity, similar to what has been observed in vitro/vivo aging models (Azman & Zakaria, 2019). These findings suggest that 25 mmol/L d‐Gal represents a threshold value for differential effects on B3p. Following 10 mmol/L d‐Gal treatment, the SO_4_^2−^ uptake rate was slowed, possibly due to A1c formation (Remigante et al., 2020), while increasing d‐Gal concentration to or above 25 mmol/L, the impact on B3p function is likely mediated by oxidative stress on a lipid and protein level.

On the basis of our results, we suggest that this in vitro model of aging induced by the d‐Gal application may give the chance to study the impact of aging at early stages, as revealed by the mechanism through which B3p transport efficiency is affected. The present in vitro model of aging shows that B3p efficiency is affected by the contribution of slight oxidative events, putatively targeting Hb. However, the involvement of oxidative stress in B3p alterations after d‐Gal treatment should not be excluded at all, as quercetin, an antioxidant interacting with cell membranes (Sangai et al., 2018), used in preliminary experiments (not shown), is able to bring the rate constant back to the control values. The involvement of Hb glycation and oxidative stress in B3p anion exchange acceleration is more clear when higher d‐Gal concentrations are used.

To better clarify the behavior of B3p after d‐Gal treatment in human red cells, B3p expression levels have also been determined (Figure 4). No change in the protein expression level was detected. This result, once again, shows that the anion exchange capability through B3p could depend on TBARS levels, alteration in the oxidative state on membrane proteins, and intracellular changes. Our previous studies reported a crosslink between B3p and Hb reflecting on the efficiency of anion exchange (Morabito et al., 2020). The affinity between Hb and B3p is characterized by a specific mechanism, where the affinity of Hb for B3p is higher than that of deoxyHb, which is the predominant form of Hb in blood microcirculation. A partial Hb oxygenation dramatically increases the rate of Hb autoxidation. This oxidizing event can damage B3p and erythrocytes membrane (Rifkind & Nagababu, 2013). The denaturation of Hb (MetHb) in the late stage of aging produces hemichromes, which have a much higher affinity for the erythrocyte membrane, thus producing an irreversible cross‐linking involving both B3p and spectrin (Ferru et al., 2011). This binding breaks the interaction between B3p and cytoskeletal proteins (ankyrin and spectrin) and triggers B3p clustering. B3p clustering increases the binding of IgG, which contributes to the removal of erythrocytes by macrophages (Ferru et al., 2011; Shimo et al., 2015). In this regard, although red cells have a life span of 120 ± 20 days in blood flow, the increased oxidative stress, involved in aging mechanisms, targets erythrocyte plasma membrane proteins and lipids, and Hb, thus altering physiological functions and reducing their life span.

In this respect, the % MetHb assay demonstrated that 24 h of incubation with d‐Gal (25, 35, 50, and 100 mmol/L) were not sufficient to induce the formation of detectable amounts of MetHb (Figure 5), while the % A1c assay showed increased glycated Hb levels (Figure 6). Therefore, in the present experimental model, A1c probably does not induce hemichromes formation or B3p clustering, but alters its cross‐link with B3p, thus leading to partial damage on anion exchange capability, as demonstrated by altered SO_4_^2−^ uptake and unchanged B3p expression levels. This result is in line with what already demonstrated by Arashiki et al. (2013), showing that both oxidative damage and clustering of B3p on the plasma membrane have been involved in the removal of senescent human erythrocytes from the systemic circulation at the end of their 120‐day life span. In this regard, both membrane peroxidation and MetHb formation were necessary for B3p clustering.

As the clustering between MetHb and B3p represents the final product of aging, which leads to erythrocytes death, and was not detected in our model, we suggest for the first time that the use of high (>25 mmol/L) d‐Gal concentrations might be useful to obtain a model of early aging. Furthermore, the presence of higher glycated Hb levels due to d‐Gal treatment reveals the formation of AGEs. These results show that oxidative stress on the erythrocyte membrane, in addition to Hb glycation, is responsible for B3p function alterations in this experimental model.

Finally, to understand how oxidative stress could affect the anion exchange capability of B3p, the endogenous antioxidant system, namely intracellular GSH, has been evaluated. The GSH/GSSG ratio after d‐Gal treatment was lower than in untreated erythrocytes, demonstrating that the antioxidant system in human erythrocytes was also altered. This result confirms that high d‐Gal concentrations have a direct effect on erythrocytes membrane and affect intracellular components, that is, antioxidant system in addition to Hb. In this regard, a recent study performed in rats reports a significant age‐related reduction in GSH levels in the brain, associated with an increase in GSH oxidation to GSSG and a decrease in the GSH/GSSG ratio (Rusu et al., 2020). In agreement with the authors, we confirm that intracellular GSH plays a pivotal role against oxidative stress in human red cells after changes induced by age‐related phenomena.

## 5 CONCLUSION

Though B3p clustering has been not investigated in the present study, an impact of early aging on erythrocytes, detected by determining B3p anion exchange capability along with glycation and oxidative events, can be recognized. Following exposure to 25 mmol/L d‐Gal, anion exchange capability is accelerated and associated with glycation of Hb, and in addition alteration of endogenous antioxidant system. By further increasing d‐Gal concentrations, SO_4_^2−^ uptake trapped amount is decreased, associated not only with glycation of Hb but also with significantly increased oxidative conditions. This means that the measurement of anion exchange capability is a sensitive tool to detect the early impact of aging on red cells. On this basis, we may conclude that: (i) d‐Gal (25, 35, 50, and 100 mmol/L) induces early aging in human erythrocytes; (ii) in the experimental model of aging induced by d‐Gal, B3p function is accelerated; (iii) d‐Gal exposure produces substantial changes on Hb and the antioxidant system; (iv) the effect of aging, mimicked by increasing d‐Gal concentrations, proceeds through steps, first involving Hb glycation and then oxidative events at the membrane level.

In this light, B3p could be considered as a potential target of antioxidant molecules to counteract aging‐related disturbances. Future experiments are needed to elucidate the signaling underlying anion exchange acceleration after d‐Gal treatment, such as the interaction between B3p and cytoskeletal proteins (ankirin and spectrin) and their potential post‐translation modifications.

## CONFLICTS OF INTERESTS

The authors declare that there are no conflict of interests.

## AUTHOR CONTRIBUTIONS

Rossana Morabito and Angela Marino conceived and designed the research; Alessia Remigante, Sara Spinelli, Vincenzo Trichilo, and Saverio Loddo performed the experiments and analyzed the data; Alessia Remigante, Sara Spinelli, Silvia Dossena, Angela Marino, and Rossana Morabito interpreted the results of the experiments; Sara Spinelli and Rossana Morabito prepared the figures; Alessia Remigante, Vincenzo Trichilo, Saverio Loddo, and Angela Marino drafted the manuscript; Alessia Remigante, Michael Pusch, Silvia Dossena, Angela Marino, and Rossana Morabito edited and revised the manuscript; Alessia Remigante, Sara Spinelli, Vincenzo Trichilo, Saverio Loddo, Antonio Sarikas, Michael Pusch, Silvia Dossena, Angela Marino, and Rossana Morabito approved the final version of the manuscript.

## Data Availability

The data that support the findings of this study are available from the corresponding author upon reasonable request.
